# Development and status of moral education research: Visual analysis based on knowledge graph

**DOI:** 10.3389/fpsyg.2022.1079955

**Published:** 2023-01-04

**Authors:** Jingying Chen, Yidan Liu, Jian Dai, Chengliang Wang

**Affiliations:** ^1^School of Marxism, Zhejiang University of Technology, Hangzhou, China; ^2^College of Educational Science and Technology, Zhejiang University of Technology, Hangzhou, China; ^3^School of Management, Zhejiang University of Technology, Hangzhou, China; ^4^Department of Education Information Technology, Faculty of Education, East China Normal University, Shanghai, China

**Keywords:** moral education, moral instruction, bibliometrics, knowledge graph, visual analysis

## Abstract

**Introduction:**

Moral education is an educational process of the continuation, construction, and transformation of moral and social norms, and is an important guarantee for the sustainable vitality of human morality.

**Methods:**

With bibliometrics applied and VOSviewer and CiteSpace as tools, this paper systematically analyzes 497 articles published in the Social Sciences Citation Index of Web of Science core collection from 2000 to 2022 in the field of moral education research.

**Results:**

By quantifying specific performance information in the field of moral education in terms of authors, journals, organizations and countries, this paper identifies the highly productive authors and organizations, as well as core journals (i.e., the *Journal of Moral Education*). A cluster analysis is used to show the knowledge structure, and an evolutionary analysis to present the macro-development trend of moral education.

**Discussion:**

In this paper, the comprehensive description of the research topics on moral education clarifies the development model and disciplinary prospect of the moral education research, and provides theoretical and practical support for the continuous development and application practice of the moral education research.

## 1. Introduction

Discussions of morality can be traced back to the ancient Greek period, when Aristotle noted in *Nicomachean Ethics* that virtue could be divided into intellectual virtue and virtue of character, and that the latter came about as a result of habit, which was people's pursuit of beauty and kindness (Ameriks and Clarke, 2000). During the more than two thousand years since then, countless scholars and philosophers have been inspired by Aristotle's *Nicomachean Ethics* and have made in-depth interpretations and explanations of morality and moral phenomena (Kristjánsson, 2006). Under the theoretical framework of Aristotelian virtue ethics, this paper attempts to classify and review the development of moral education in K−12 and higher educational systems.

Moral education is a grand concept that involves many disciplines (MacIntyre and Dunne, 2002; Kristjánsson, 2021). Generally, the essence of moral education is the process by which educators transform certain social thoughts and *virtue ethics* concepts into the individual thoughts and morals of educatees with certain educational means in social activities and exchanges (Solomon et al., 2001). Thus, moral education is mainly the process of moral social inheritance or transmission.

Different from disciplinary education, the value of moral education in practice has been controversial (Peters, 2015). Some scholars have questioned the necessity for schools to provide moral education (Stanley, 2003, 2004; Motos, 2010); however, more scholars have agreed that schools should supply systematic moral education and have provided corresponding bases for doing so (Hoekema, 2010; Wong, 2020; Sison and Redín, 2022). These scholars have considered that schools have the responsibility and obligation to help students contribute to society in ways that are not limited to the value of social production but that also consider the prosocial value of promoting social goodness from the moral perspective (Hoekema, 2010). Meanwhile Sison and Redín (2022), based on MacIntyre's moral education principle, emphasized the importance of moral education in educational institutions, as an “intrinsic value of an educational institution that instills virtues … [schools should] provide ethical training on par with scientific-objective and technical training” (Sison and Redín, 2022; p. 13). Undoubtedly, these disputes have deepened the value and connotation of moral education and have established a close connection between moral education and other disciplines (e.g., business education), which efforts have increased the value placed upon moral education by scholars (Lee, 2022). At the same time, the in-depth thinking and scholarly refutation has vigorously promoted moral education studies, transforming the discussion from the necessity of moral education to its contents and purpose.

The battle has been long and arduous for moral education to play an important role in public schools. However, thanks to the efforts of scholars, moral education has become an indispensable part of school education (Leihy and Salazar, 2016). Nevertheless, disputes remain on how to implement moral education as well as on its connotation and value (Wong, 2020; Lee, 2022). The differences remain unclear in the moral educational issues in terms of cultural environments and social backgrounds, and systematic and comprehensive quantitative reviews and analyses are lacking in the moral education literature. Therefore, this paper aims to apply the method of a literature review to systematically organize and further analyze the research on moral education in K−12 and higher educational systems after a comparison and an identification, mainly focusing on the following points:

1. We conduct a systematic performance analysis of the research topics on moral education; know the authors, organizations, and countries with high productivity in the field of moral education; and thoroughly uncover the main journals and highly cited studies in this field.
2. We reveal the core issues and research status in the field of moral education through a cluster analysis and summarize the research results.
3. We provide theoretical and practical support for the subsequent academic research and practice of moral education using evolutionary and keyword-burst analyses to delineate the evolutionary trend of the field.

## 2. Literature review

Morality can be traced to the origin of human language. In exploring the origin of morality, Tappan (1997) proposed that morality, as a high-level psychological function, was mediated/regulated by the forms of words, language, and discourse. Per Tappan, as language is a remarkable social medium, the process of social communication and social relations inevitably produce moral function. Tappan also argues that because words, language, and discourse forms are essentially social and cultural phenomena, moral development has always been affected by the specific social, cultural, and historical background in which it occurs.

Morality, as a uniquely human higher mental function, has long been noticed by scholars. In ancient Greece, Socrates incorporated the study of moral ethics into the philosophical system and created his own “philosophy of ethics.” Aristotle further wrote *Nicomachean Ethics*, which describes the qualities of an ideal or perfect human being: courage, temperance, generosity and magnificence, and possessing a great soul (Ameriks and Clarke, 2000). Aristotle provided the most basic definition of virtue ethics, which is considered the systematic origin of virtue ethics (Ferrero and Sison, 2014). The morality research has been continuous as human civilized has evolved. For example, Aquinas in the Middle Ages and Machiavelli in the Renaissance built ethical discourse systems (McInerny, 1997; Bielskis, 2011). However, due to social and historical limitations, the past research on morality has mostly relied on experience, and scholars have mostly discussed morality from the theoretical or philosophical level. Not until the psychologist Wundt established the first psychology laboratory (in 1879) did scholars begin to use modern scientific research methods to discuss morality. Soon thereafter, the research on moral education reached a development peak.

Piaget (1932) put forward Piaget's Theory of Moral Development based on his observation on children's play, initiating the scientific and systematic research on moral education (Peters, 2015). Based on Piaget's research, as well as that of Dewey (1959) and others, Kohlberg (1969, 1973) proposed a more valuable moral theory, namely, that of moral cognitive development, which was later revised and improved. The theory of moral cognitive development states that moral education is intended to help young people learn to justify moral claims correctly and rationally and to develop logical strategies to draw correct inferences from such claims when dealing with moral dilemmas (Kohlberg, 1981). Kohlberg's theory attracted great attention in sociology and psychology, and it aroused intense discussion (Mischel, 1971; Lickona, 1976). Kohlberg's theory was partially overturned in subsequent empirical studies (Kuhn, 1976). Nevertheless, as the first systematic and complete theory of moral cognitive development, Kohlberg's theory of moral cognitive development has made an indelible contribution in promoting people's cognition of morality and has successfully caused many scholars to focus on moral education.

The value of Kohlberg's theory of moral cognitive development rested not only in the theory but also in his research method, which provided a perspective for an in-depth understanding of the development of moral thinking. However, because the research design was not entirely rigorous, for example, the subjects used were all male (Aron, 1977), the theory also received some criticism and spawned further studies (Gilligan and Attanucci, 1988; Rest et al., 1997), causing the research on moral development to present a diversified development trend.

The criticism of Kohlberg's moral theory and its development were accompanied by the beginning of the theories of constructivism and humanism. Humanistic theory, in particular, positively affirmed humanity and considered that human nature is kind, rational, positive, and trustworthy. The theory proposes that moral education is required because human environment after birth has many bad factors that hinder the development of human nature's innate potential. However, the basis of moral education is rational, positive, and active humanity, a theory upon which many Chinese and Western scholars have reached an agreement (Slote, 2016).

Societal development and changing times have endowed the moral education research with new elements. In the 1980s, a systematic moral education curriculum system emerged in many region's schools (Cheung and Lee, 2010). However, the initial practice of moral education was a process of exploration, and the development process was accompanied by many frustrations. For example, in the late 20^th^ century, many scholars criticized the excessive emphasis placed on moral skills in the process of traditional moral education (Doyle, 1997; Lickona, 1999). These scholars put forward a new concept of character education to emphasize the specific content (a set of specific values) behind morality: trustworthiness, respect, responsibility, honesty, justice, and fairness (Berreth and Berman, 1997; Fenstermacher, 2001).

Since the 21^st^ century, the frequent contact among different cultural groups has added a multicultural perspective to moral education. Some scholars have noted that the main goal of moral education is to achieve equality between different groups and allow them to maintain contact with the overall culture of society (Ranson, 2000). Therefore, moral education practitioners should teach students communication skills. Some scholars have also noted and emphasized that moral education should create channels for learners to understand society's diversity (Banks et al., 2001). That is, moral education should cultivate learners with a broad cultural vision and cultural inclusiveness (Santas, 2000). This suggestion means that the historical and cultural perspectives of different social groups should be included in the moral education curriculum (Kumashiro, 2000).

Meanwhile, as the concept of a postmodern society spreads, moral education development has transformed from a discipline that emphasizes the standardization and objectivity of rationality and science to one that pays attention to educational value, diversity, and context (Sarid, 2012). In this process, the moral education research method, contents, and objects have undergone profound changes. For example, the speculative reasoning research has gradually been replaced by the empirical situation research, and moral education has begun to emphasize the emotional commitment and developmental reflection made by individuals in the growth process (Wardekker, 2004). Civic and value education have been gradually incorporated into the category of moral education and have become an indispensable part of it (Schuitema et al., 2008). Finally, the research objects have gradually expanded from learners to practitioners of moral education and school administrators (Reiman and Dotger, 2008). Meanwhile, diversified education has put forward some new standards for moral education. For example, moral education should pay more attention to learners' personality factors than to disciplinary education, including social identity factors consisting of race, gender, and class and personality factors such as character and temperament (Schuitema et al., 2008). Therefore, the mission of moral education has gradually come to include social identity construction.

The discipline systematization of moral education is also an overall trend of the development of moral education (Zhang et al., 2022). Increasingly, scholars have begun to discuss subject-specialization for moral education and standardizing the curriculum design (Bleazby, 2020). In addition, the school ages and stages related to moral education have also been expanded. Some scholars have proposed that the cognition of moral education should not be limited to the moral training received at school, and moral education should become a part of the lifelong learning process (Higgins-D'Alessandro, 2011; Wong, 2020).

The rapid development of postmodern technology has expanded the new dimension of moral education, such as defining the moral norm in the environment of mass media and networking (Internet) and how to implement the corresponding moral education (Wanxue and Hanwei, 2004; Li et al., 2017; Chang et al., 2018). Technology is a double-edged sword. On the one hand, the openness, anonymity, and interactivity of the Internet are challenging the traditional moral concept, especially the college students who are widely exposed to online we-media are faced with largescale moral anomia (Li et al., 2017; Shao and Wang, 2017). On the other hand, based on the application of human-computer interactions and virtual reality scenes, artificial intelligence can achieve a more realistic situational experience of moral education. Regarding the hidden concern that artificial intelligence might replace teachers in moral education, current scholars have a relatively consistent view that human teachers in moral education will be irreplaceable for a long time (Tan, 2020).

Summarizing the current research on moral education, its purpose in schools is to prepare students to participate in society (Schuitema et al., 2008), but this purpose is not isolated. Instead, it can be divided into two supportive aspects: firstly, its aim is to serve students' individual development to guide students to adulthood where they can produce their own social identity; secondly, moral education hopes to promote the rational, orderly development of society by cultivating students' prosocial behaviors, as viewed from the social development perspective. These two aspects of moral education reflect two perspectives on it (personal and social). In fact, these two perspectives complement each other and together constitute the profound connotation of moral education. These underlying connotations do not change dramatically over time, showing that morality is uniquely stable in the tide of diversification and modernization. However, the multiple dimensions of culture and the rapid development of technology continually call into question the implementation and practice of moral education. In response, we must deeply examine this era and learn the development course and the current discipline structure of moral education.

## 3. Methods and materials

### 3.1. Research method

To understand the research agenda of moral education systematically, objectively, and comprehensively from a global perspective, this paper adopted a bibliometrics approach for the analysis. Bibliometrics is a measurement method used to describe and analyze the dynamics and progress of a discipline or research field. Since 1969, when British scholar Pritchard put forward “bibliometrics,” as an independent discipline, it has become prominent in scientific quantitative research. Meanwhile, benefiting from the recent developments in computer science and technology, econometric analyses combined with visual analyses have become a new trend in this research field. Econometric analytical results can be displayed in simple and clear knowledge graphs, thus achieving the goal of “one picture is worth ten thousand words” (Merigó et al., 2015).

In this paper, CiteSpace 6.1.R3 (developed by Chen C. at Drexel University), VOSviewer 1.6.17 (developed by Van Eck and Waltman at the Center for Science and Technology Studies) and SCImago Graphica 1.0.24 (developed by Scimago Lab in Spain) were used to draw knowledge graphs. Each software package has its own advantages, and together they can play complementary roles. CiteSpace adopts the data standardization method based on set theory to measure the similarity of knowledge units. By drawing a Timezone view, CiteSpace can clearly outline the evolutionary process of research hot spots in the temporal dimension, thus presenting the development process and trend of this field (Wang et al., 2022). VOSviewer adopts the data-standardization method based on probability theory and provides a variety of visual presentations of keywords, co-organizations, co-authors, etc. With simple drawings and elaborate images, at present, it has increasingly attracted scholars' attention in the visualization field of bibliometrics (Pan et al., 2018). SCImago Graphica, on the other hand, can use table data in various formats exported from CiteSpace and VOSviewer for redrawing to supplement the mapping.

### 3.2. Initial literature search

In the initial literature search of this paper, the Web of Science core collection was mainly used. This was because many review studies have posited that the literature quality of the data source is crucial to the reliability and persuasiveness of the review study (Hwang and Tsai, 2011; Hsu et al., 2012). As a high-quality digital literature resource database, the Web of Science core collection has been accepted by many researchers (Ding and Yang, 2022). Within this collection, the Social Science Citation Index (SSCI) is the most well-known journal index in the field of social sciences (Yadegaridehkordi et al., 2019). Taken together, these methods guarantee the quality of the literature used in this paper.

Literature retrieval is also an important link to ensure research quality. Since some scholars (Ferrero and Sison, 2014) have tried to review virtue ethics in business by means of quantitative reviews, this paper follows the model of Ferrero and Sison (2014) in the literature retrieval.

When setting the retrieval strategy, only the advanced retrieval function of Web of Science core collection is used in this paper. The input was the searchable TS = (“moral education” OR “moral instruction”), and Topics (TS) was used as the Field Tag to implement searchable matching in the title, abstract, and other informational elements of the literature. Such a search strategy can retrieve the literature related to moral education as comprehensively as possible. At the same time, to ensure sufficient data to analyze the development trend of the research topics on moral education, the selected literature search period was January 2000 to September 2022, and the literature types Article and Review were selected. The retrieval-based search resulted in 842 articles, the specific information for which is shown in Table 1.

**Table 1:** Summary of data source and selection.

**Category**	**Specific standard requirements**
Research database	Web of science core collection
Citation indexes	Social science citation index (SSCI)
Searching period	January 2000 to September 2022
Language	“English”
Searching keywords	TS = (“moral education” OR “moral instruction”)
Document types	Articles and Reviews
Sample size	842 (Before Screening)

### 3.3. Literature screening

The literature obtained was often mixed with some irrelevant results (e.g., literature taking moral education as a research background but analyzing other research contents). Therefore, to ensure that the literature included in the analysis was closely related to the relevant topics, software was required to remove and manually screen the literature included in the analysis after the initial literature search. Doing so prevents the analytical results from suffering due to data quality problems (Chen et al., 2022).

The “remove duplicate” function of CiteSpace software was used to discard two duplicates and proceed to the manual screening. To ensure a scientific and reliable screening process, this paper refers to the literature screening criteria proposed by Su et al. (2019). Before screening, the three team members consulted with moral education scholars to determine the inclusion and exclusion criteria. This standard was mainly to review the core research contents and themes, to identify the primary focuses in the research related to moral education in K−12 and higher education (such as the implementation path of moral education, the influence factors of the moral education effect, etc.), and to exclude some articles whose research subject was not moral education or whose research field was not within the scope of K−12 and higher education.

After determining the inclusion and exclusion criteria, the three team members independently reviewed each article according to the criteria. In the case of controversial articles, discussions and votes were held to decide whether to exclude them.

After this systematic screening process, 342 articles were deleted as they did not meet the requirements of this study, and 497 articles were ultimately retained for further analyses. VOSviewer was used to collect basic statistical information on the selected literature. These 497 articles originated from 426 organizations in 49 countries, had 759 authors, were published in 132 journals, and cited 16,815 references from 10,648 journals.

## 4. Performance analysis: Productivity and impact

### 4.1. Publication time trend

**Figure 1:**
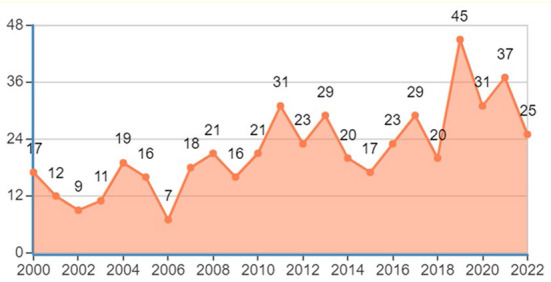
Time trend of the publications on moral education.

To understand a research field, it is necessary to first understand the most basic quantitative information, among which, the change in the annual publication number can best reflect the development trend of a research field. Figure 1 shows the temporal distribution of papers published in the moral education research. Overall, the publication number in this field is still increasing although fluctuating and not obvious. In 2000, the number of published articles reached 17, indicating that the moral education research has been active for a long time, rather than being a new topic. Additionally, in the past 5 years, the number of published articles was 20+, indicating that this topic has not declined gradually over time but has evolved continually as the classical scholars' thoughts and views are constantly reflected upon, reshaped and extended (Lewis, 2018; Hand, 2019), and the topic remains vital as an independent discipline.

### 4.2. Authors

By analyzing the number of articles authors publish, we can learn the representative scholars and core research topics in the moral education research. This paper used Price's law to calculate the boundary between ordinary and core authors in this field:


$$\begin{array}{l} m\;=\;0.749\times \sqrt{n_{\max}} \end{array}$$

where *n*_max_ is the number of papers by the most productive authors in this field (*n*_max_ = 11 according to VOSviewer's statistical analysis), and *m* is the minimum number of papers by the core authors, which can be calculated as *m* ≈ 2.5. Therefore, authors with ≥3 papers were identified as the core authors in this field (Price, 1963), and there were 20 core authors. Table 2 presents the relevant information on the core authors in this field, including their names, the number of published articles, and the citation number per article.

**Table 2:** Core authors in the moral education research field.

**Rank**	**Author**	**Counts**	**Citations per paper**	**Rank**	**Author**	**Counts**	**Citations per paper**
1	Han, H.	11	17.09	11	Swartz, S.	4	4.75
2	Kristjánsson, K.	9	14.89	12	Thoma, S. J.	3	9
3	Huo, Y.	4	1.25	13	Sanger, M. N.	3	45
4	Thornberg, R.	4	38.25	14	Dawson, K. J.	3	5.33
5	Xie, J.	4	1.25	15	Meindl, P.	3	10.33
6	Osguthorpe, R. D.	4	30	16	Akar, H.	3	7.67
7	Kuusisto, E.	4	6.25	17	Carr, D.	3	28.33
8	Tirri, K.	4	6.25	18	Sen, D.	3	7.67
9	Nucci, L.	4	32	19	Temli, Y.	3	7.67
10	de Ruyter, D. J.	4	10.25	20	Veugelers, W.	3	6.33

Table 2 shows that Han is the most productive author in this field. He has published 11 papers in the past 20 years. Han and his research team have mainly focused on moral exemplars in moral education (Han et al., 2022). The moral exemplars in teaching materials and voluntary service have been deeply studied (Han et al., 2017, 2018b). Moreover, Han is a pioneer in advocating technology-enabled moral education. Several of his studies have used Bayesian models to predict the relationship between moral foundations and the development of moral judgments (Han et al., 2018a; Han and Dawson, 2022). Kristjánsson's scientific productivity in the field of moral education is second only to Han's. Kristjánsson is a classical scholar who paid great attention to Aristotle's thought. Many of Kristjánsson's studies have focused on the value of Aristotle's thought in contemporary moral education (Kristjánsson, 2014, 2020), proposing that the wisdom of classical philosophers should not be ignored in contemporary moral education, and implementing a practical and critical inheritance of Aristotle's philosophical thought (Kristjánsson, 2013). Kristjánsson is also the editor-in-chief of the core journal, *Journal of Moral Education*, in this field (Kristjánsson, 2021).

### 4.3. Journals

Journals are the main carriers of literature. This paper performs statistical analyses of the journals that publish moral education research (Table 3 shows the top-10 core journals in terms of published article volume). The results show that most of the research results in this field were published in the *Journal of Moral Education* (198, accounting for 39.76% of the total), while the second-ranked journal published only 34 research papers on moral education (approximately 6.83% of the total). Regarding the distribution of the published article volume, the Matthew effect was significant because moral education is highly focused and independent. In addition to the *Journal of Moral Education*, a journal closely related to moral education, other journals that focus on moral education are mostly related to educational philosophy (such as the *Journal of Philosophy of Education, Educational Philosophy and Theory*, and *Studies in Philosophy and Education*). This finding shows the close relations among moral education, educational philosophy, and virtue ethics. In addition, from the perspective of the average citation frequency, *Teaching and Teacher Education* had a high citation frequency (31.67 times on average), indicating that teacher education is highly relevant to the moral education research (Xiaoman and Cilin, 2004).

**Table 3:** Top 10 journals in the moral education research field.

**Rank**	**Journal title**	**Counts**	**Citations**	**Citations per paper**	**Impact factor (2021)**
1	Journal of Moral Education	198	2,232	11.27	1.398
2	Journal of Philosophy of Education	34	214	6.29	0.949
3	Educational Philosophy and Theory	27	174	6.37	2.054
4	Teaching and Teacher Education	15	475	31.67	3.782
5	Journal of Beliefs and Values-Studies in Religion and Education	11	56	5.09	1.724
6	Studies in Philosophy and Education	7	42	6	1.629
7	British Journal of Religious Education	6	112	18.67	1.131
8	Journal of Curriculum Studies	6	89	14.83	2.175
9	Educational Review	6	73	12.17	3.829
10	Asia Pacific Journal of Education	6	41	6.83	1.478

### 4.4. Countries and organizations

**Figure 2:**
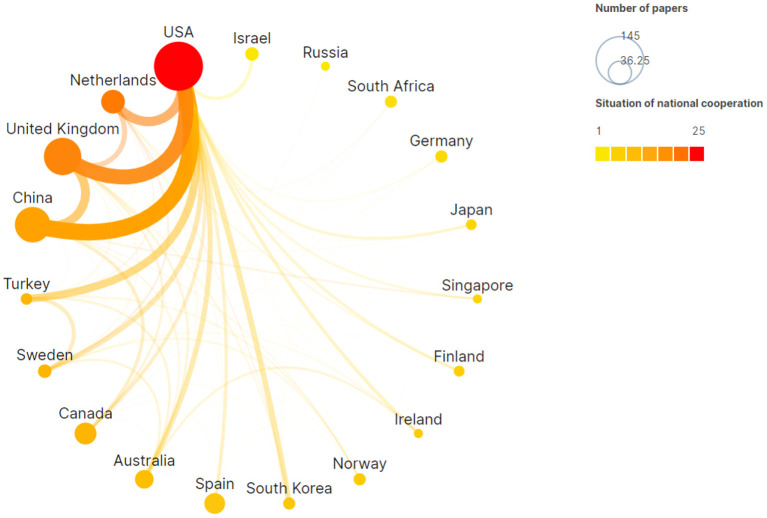
Chord graph of inter-country number of papers and cooperation.

An analysis of the countries in which the research was published can reveal the countries and research organizations with high productivity in this field. To have a clear understanding of the number of publications and cooperation situation between different countries, this paper used a chordal graph for elaboration. Chordal graphs are mainly composed of nodes and chords. Nodes represent the number of certain country's published articles and are arranged along the circumference and in a radial series. The node colors represents the cooperation intensity with other countries, and colors closer to red indicate greater more cooperation with other countries. An arc with a weight (and a width) connecting any two nodes is called a chord, which represents the between-country cooperation. The resulting chord graph of the intercountry number of publications and the cooperation network is shown in Figure 2.

Figure 2 shows that the main countries with large publication numbers and intercountry cooperation in this field are the USA, the United Kingdom, China and the Netherlands. These four countries not only publish a large number of articles but also cooperate closely. They have close international academic exchanges and a high degree of internationalization in the moral education academic research. Table 4 gives more specific quantitative information for the top-10 countries in terms of publication number. Except for China, the remaining 9 countries are all developed countries, indicating that moral education is an issue that many scholars pay attention to only after a society develops to a certain degree and has a certain economic foundation.

**Table 4:** Top 10 countries in the moral education research field.

**Rank**	**Country**	**Counts**	**Citations**	**Citations per paper**
1	USA	145	2,035	14.03
2	United Kingdom	84	981	11.67
3	China	76	535	7.04
4	Netherlands	34	383	11.26
5	Canada	29	267	9.21
6	Spain	26	161	6.19
7	Australia	21	223	10.62
8	Sweden	11	289	26.27
9	Israel	11	45	4.09
10	Norway	10	92	9.2

A further analysis was made of the issuing organizations. Table 5 shows the top-10 organizations in terms of publication number and their related quantitative information. Among these organizations, most (up to 5) are from the United Kingdom, among which the University of Birmingham is the most productive organization, with 17 published articles, making it the primary academic force in the moral education research. Most of this organization's articles were published between 2014 and 2020 and focused on philosophical discussions of moral education, many of which tried to relate the thoughts of ancient Greek philosophers and use them as guidance to carry out moral practice (Carr, 2014; Jordan and Kristjánsson, 2017). Stanford University, located in the USA, has both a high publication and a high citation number (35 times on average), mainly due to Noddings (2010), Han et al. (2017, 2018a,b, 2022), and Han and Dawson (2022).

**Table 5:** Top 10 organizations in the moral education research field.

**Rank**	**Organization**	**Country**	**Counts**	**Citations**	**Citations per papers**
1	University of Birmingham	United Kingdom	17	194	11.41
2	University of Alabama	USA	10	109	10.9
3	Vrije University Amsterdam	Netherlands	10	147	14.7
4	University of Edinburgh	United Kingdom	8	132	16.5
5	University of Nottingham	United Kingdom	8	25	3.13
6	Stanford University	USA	7	245	35
7	University of Cambridge	United Kingdom	7	37	5.29
8	University of Oxford	United Kingdom	7	156	22.29
9	National Taiwan Normal University	China	7	53	7.57
10	Hong Kong University of Education	China	7	14	2

### 4.5. Articles

Highly cited articles can often reveal the key issues and core points of differentiation and analysis in a research field. The highly cited articles in the moral education are shown in Table 6. The most frequently cited study from 2000 to 2022 was Villenas (2001), a qualitative study on family moral education that analyzed the key role of mothers in family moral education from the perspective of feminism and antiracism through interviews with many Latino mothers. The second most frequently cited was a speculative study by Halstead (2004), which systematically analyzed moral education in Islam from its basic philosophical issues. A review of the highly cited articles further reveals that moral education is a very broad topic. These highly cited articles cover many aspects of moral education, such as teacher (Sanger and Osguthorpe, 2011), ethics (Woods, 2005), and value education (Thornberg, 2008). Other scholars have systematically discussed how moral education balances the threats and sense of alienation created by technological development (Persson and Savulescu, 2013). Moral education is thus not only related to the words and deeds of each individual but also closely related to social groups. Meanwhile, to present the articles in the moral education research more comprehensively and three-dimensionally, this paper identified the remaining 90 among the 100 articles with the highest citation frequency from 2000-2022 (see Appendix 1 for details).

**Table 6:** Most cited articles between 2000 and 2022.

**Rank**	**References**	**Title**	**Year**	**Citations**
1	Villenas (2001)	Latina mothers and small-town racisms: creating narratives of dignity and moral education in North Carolina	2001	124
2	Halstead (2004)	An Islamic concept of education	2004	110
3	Hardy and Carlo (2005)	Identity as a source of moral motivation	2005	94
4	Thornberg (2008)	The lack of professional knowledge in values education	2008	84
5	Persson and Savulescu (2013)	Getting moral enhancement right: the desirability of moral bioenhancement	2013	84
6	Nucci and Turiel (2009)	Capturing the complexity of moral development and education	2009	83
7	Sanger and Osguthorpe (2011)	Teacher education, preservice teacher beliefs, and the moral work of teaching	2011	78
8	Chouliaraki (2008)	The media as moral education: mediation and action	2008	76
9	Noddings (2010)	Moral education in an age of globalization	2010	70
10	Kristjánsson (2006)	Emulation and the use of role models in moral education	2006	64

## 5. Keywords analysis: Cluster, evolution, and burst

### 5.1. Keyword co-occurrence analysis

**Figure 3:**
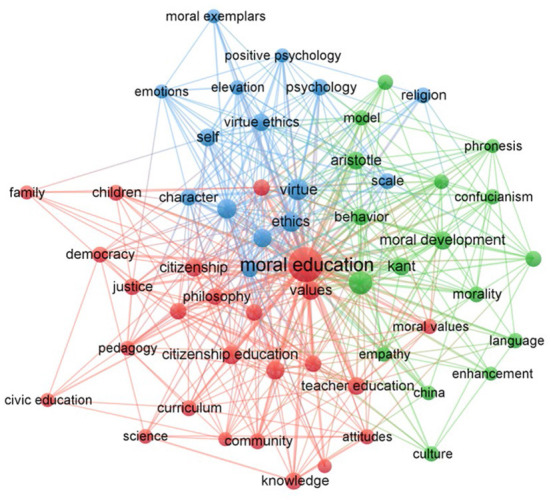
Co-occurrence of keywords.

Keywords condense an article's core and essence. Research hot spots in a certain field can be found through keyword co-occurrence analyses, so keywords have been widely used to reveal the knowledge structure of the research field (Chen and Xiao, 2016). In this paper, VOSviewer was used to visualize high-frequency keywords and display those with frequencies > 5. The results are shown in Figure 3. In the keyword co-occurrence knowledge graph, the node sizes reflect the keyword frequency: larger nodes indicate that the keywords appeared more frequently. The node colors represent different clusters, namely, the research topics. The lines between the nodes represent the strength of association: thicker lines indicate that the keywords appear more frequently together in the same article.

As can be seen from Figure 3, the moral education research has three main clusters. To learn the research details of the focus within each cluster, the following 3 clusters are analyzed individually.

In the blue cluster, the research studies moral education from the psychological perspective. Many studies have focused on the effect of moral exemplars (Han et al., 2017, 2018b). Studies have also examined how the psychological levels are associated with moral development, including self-preservation (Dahlbeck, 2017), self-doubt (Verducci, 2014), and self-cultivation or self-shaping. Such studies explain the value of morality and moral education from the perspective of psychology. Other psychological studies have measured the motivational strength of moral behavior by the scientific measurement method (Bock et al., 2021). Some studies have also analyzed the kind of moral education that should be given from the perspectives of belief and religion (Lin and Lu, 2020). In addition, virtue ethics is a focal point covered by this cluster and has attracted much attention in the field of moral education. Virtue ethics has been deeply discussed by many scholars; for example, advocates of virtue ethics have launched a heated debate on whether a shared public moral education system is possible (Katayama, 2003).

Research in the green cluster focuses on the more specific critical thinking and practical research on moral education. As a discipline derived from educational philosophy, many studies on moral education still follow the discourses and speculative research methods used in the philosophical research (Nakazawa, 2018), for example, by comparing the thinking and practical models of scholars such as Kant, Aristotle and Mill and by discussing their contributions to moral education (Surprenant, 2010). Cross-cultural comparative analyses and speculative studies are also an important component of this cluster and have become an important model for the creation of new thoughts on moral education. Some scholars, after learning the shortcomings of Western educational philosophers' thoughts, began to promote the Oriental Confucian view of moral education (Sin, 2022). Some scholars have tried to explain whether the effect of moral education was internally or externally driven by comparing the thoughts of famous thinkers in the field of moral education between China and the West (Slote, 2016). In addition to the speculative research, which is more profound, many comparative empirical studies in this cluster have focused on moral practice (Chi-Hou, 2004; Cheung and Lee, 2010; Ronkainen et al., 2021). For example, Asif et al. (2020) compared the differences in the training objectives of moral education between Chinese and Pakistani teachers by combining qualitative and quantitative research methods. The teachers in Pakistan considered the sovereignty of sacred laws, loyalty to the country's constitution, and a sense of service to society as the ultimate goals of a moral education. Meanwhile, the Chinese teachers promoted a political ideology that stressed collectivism in a socialist approach, with family and social values being the most relevant. As moral education is a research topic involving social culture, historical background, and temporal characteristics, discussions on its object and implementation method are quite complicated. The research in the green cluster tries to reveal the complex relationships from more abstract ideological discussions and a more concrete empirical analysis to delineate the big picture of the moral education research.

The research in the red cluster mainly studies civic education, which is a very important subtopic in moral education. Although some scholars have proposed that moral education should be distinguished from civic education (Cantero, 2008), many scholars have reached a consensus on this issue at present. Civic education is believed to be a research topic under the general concept of moral education (Schuitema et al., 2008). This status is because the essence of moral education on the social level is to promote the orderly and rational development of society by cultivating students' prosocial behaviors. Therefore, all democratic societies should pay attention to citizen socialization, that is, for everyone in a democratic society to know their citizenship status. Moral education plays an important role in this process (Althof and Berkowitz, 2006). Obviously, the social meaning of moral education is highly consistent with civic education. However, the use of civic education to replace moral education is not comprehensive, and some scholars have noted problems in talking about moral education only from the social level. In a democratic society, it is necessary to constantly weigh the balance between the advantages and disadvantages of public rights and private rights, requiring teachers engaged in the work of moral education teach social expectations for qualified citizens on the one hand, and citizens develop the self-awareness and moral awareness, on the other hand (Bernal Guerrero et al., 2019).

The cluster analysis of moral education shows that the main research schools at present are moral psychology, moral education philosophy, and civic education. However, a careful examination of the moral bases followed by these schools shows that they cannot be separated from Aristotle's framework of moral virtues. The moral psychology schools are mostly based on the virtue theory of positive psychology (Seroczynski, 2015). The moral education philosophy schools are also based on the derivation of Aristotelian concepts such as morality and virtue (Surprenant, 2010). Civic education regards the establishment of certain sociopolitical mechanisms as a prerequisite for maintaining moral education (Carr, 2006; Kristjánsson, 2014), which also coincides with some ideas discussed by Aristotle. Therefore, a consistent tradition and inspiration in the moral-related and moral education research for many years has been the inspiration taken from the ancient philosopher Aristotle's thought. It acts much like a towering tree: many research schools have undergone steady development and growth but remain firmly rooted in the thought foundation of ancient philosophers.

This cluster analysis of moral education also shows that it is a complex multidimensional and interdisciplinary topic, involving pedagogy, psychology, sociology, philosophy, and other fields (Chi-Kin Lee et al., 2021). People from different disciplines have different opinions on moral education (Alvey, 2001). Each disciplinary perspective provides an indispensable piece of the jigsaw puzzle that is the overall picture of moral education. In addition, the cluster research demonstrates a typical characteristic of the moral education research—the emphasis placed on theoretical research and analysis—which is due to the subject's particularity. Until the present, mainly educational philosophers have made profound analyses of moral education, and educational philosophers have often chosen to develop the field of moral education by argumentation (Lewis, 2018). Regarding the discipline's development, the emergence and initial development of any discipline depends on high-quality argumentation to realize the conceptualization and categorization of the discipline's terms, and the same is true of moral education. These wonderful arguments are difficult to reflect in this paper in terms of a simple and concise conclusion, but they are the objects worthy of appreciation. Therefore, the cluster analysis presents only the overall style of moral education, and the brilliant internal testimony and argumentation among scholars requires readers to examine the classical literature carefully. Moreover, the cluster research also finds that under the influence of positivism, all kinds of empirical studies in moral education have increased in recent years, and the use of qualitative and quantitative analytical technology has enriched the research model of this issue. Undoubtedly, the introduction of the empirical paradigm endows this topic with scientific nature, extends the scope of the moral education research, and expands the value of moral education as a separate discipline.

### 5.2. Keyword evolutionary analysis

**Figure 4:**
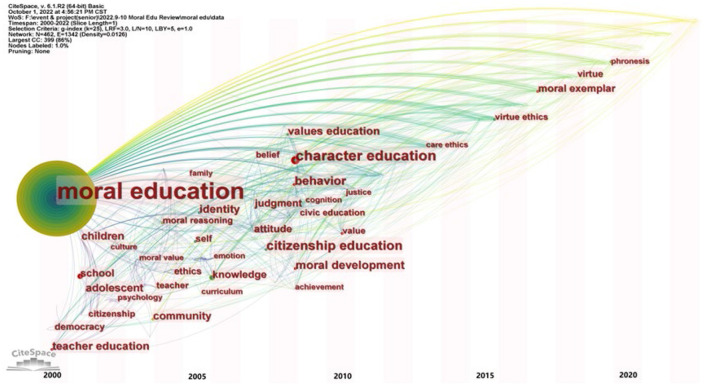
Time zone diagram of evolution of keywords in moral education research (keywords with frequency >5).

Keyword co-occurrence analyses can reveal the hot spots and focal points of research fields, thus showing the structural characteristics of moral education issues and the development process of the research field. In this paper, CiteSpace was used to conduct an evolutionary analysis and delineate the view of keyword time zones (see Figure 4). In Figure 4, each background bar in the time zone diagram represents a year, the keyword size represents the keyword frequency, and the line represents the keyword co-occurrence relationship.

Figure 4 shows that the high-frequency keywords essentially appeared before 2010, and only 4 keywords with frequencies > 5 appeared after 2015. This result indicates that the concept development of moral education has entered a relatively stable stage in recent years, and the discipline structure tends to be perfect. Therefore, the evolutionary analysis should focus on the development of and change in keywords from 2000 to 2010.

At the beginning of the 21^st^ century, the subdomain of moral education, including topics such as its teachers and mission (Fallona, 2000; Wardekker, 2001; Husu and Tirri, 2003), was widely studied. In addition, some research topics, such as democracy and citizenship, were more specific and reflected scholars' expectations about moral education's goals, including understanding and recognizing obligations and responsibilities in a democratic society, seeking equality in human rights, and understanding the connotation of citizenship (Brabeck and Rogers, 2000). The keywords “children” and “adolescent” reveal the main groups with which moral education was concerned at that time (Kuther and Higgins-D'Alessandro, 2000). This focus is different from the view that “moral education is an integral part of lifelong education,” held by some scholars in recent years (Wong, 2020). This difference shows that the generalization in moral education's object has been a major trend in the past 20 years. At the same time, moral education in the early 21^st^ century was more about the value and benefit of the individual educatee (Wardekker, 2001). Some studies paid attention to the value of moral education in improving adolescent self-esteem (Covell and Howe, 2001). However, the research at that time paid less attention to the larger social benefit of moral education. To some extent, this absence affected the construction of moral education's goal, making it slightly one-sided and narrow.

Meanwhile, the wide opportunities for moral education in schools at all levels has triggered scholars' systematic research on moral education at the instructional design level (Jie and Desheng, 2004). During this period, the curriculum and teaching theory system have been constructed belonging exclusively to moral education (Tai Wei and Lee Chin, 2004). At this time, the curriculum orientation of moral education was based on the pursuit of personal wellbeing and citizens' moral qualities (Lee and Ho, 2005). Many studies have attempted to guide the development of the moral education curriculum based on previous theoretical studies (Jie and Desheng, 2004; Richmond and Cummings, 2004). The reform of the moral education curriculum has become a new hot spot, as reflected in “curriculum” and other keywords in Figure 4. At this time, under the guidance of Chinese government policies, Chinese scholars' research has become the forefront of curriculum theory in moral education, and has put forward much practical guidance for the curriculum system design of moral education (Chi-Hou, 2004; Wansheng and Wujie, 2004; Lee and Ho, 2005; Cheung, 2007).

Between 2008 and 2012, moral, value, character, and civic/citizenship education have significantly and increasingly diverged, becoming emerging hot spots for scholars (Gilead, 2011). Civic education particularly compensates for the drawbacks of the past moral education that focused too much on individual values, and it emphasizes the social benefits of individual identification with citizenship (Schuitema et al., 2008). In contrast, value education, in its emphasis on the construction of learners' values, together with moral education, involves the specific connotation of moral education at the individual level (Marshall et al., 2011; Pantić and Wubbels, 2012). As moral education continues to differentiate, concrete research has begun to increase, since the detailed issues require the support of the micro-empirical research. Although the abstract and philosophical speculation and argument remained the mainstream research trend, they were no longer in a monopolistic position.

The empirical paradigm and hybrid research methods widely used in discipline education and higher education have also been used by the moral education researchers (Dahlin, 2010). This fact has become a turning point that cannot be ignored in the process of promoting moral education's development.

Overall, moral education since 2012 has essentially continued its past development trend, with few typical signs of discipline development and evolution. Although the outbreak of COVID-19 has reshaped the model of contemporary education, it seems to have had little impact on moral education at the academic research level. Until now, no scholars have systematically discussed the impact of COVID-19 on moral education. This is a blank area that the current research on moral education needs to pay attention to, because there is no doubt that the great changes in society will bring multidimensional challenge to moral education and promote its deeper reflection and development.

Moreover, the current development and evolutionary situation of moral education have also attracted scholarly attention. Krettenauer (2021) noted that in the social sciences and related fields, the morality research increased exponentially in the past 15 to 20 years, but the moral education research had not seen a corresponding upsurge. However, unfortunately, Krettenauer (2021) also failed to reveal the mechanism behind the phenomenon, and therefore failed to make constructive suggestions to resolve it. Perhaps this is also a specific research direction under the larger topic of moral education that still has present research value and requires further scholarly exploration.

### 5.3. Keyword-burst analysis

**Figure 5:**
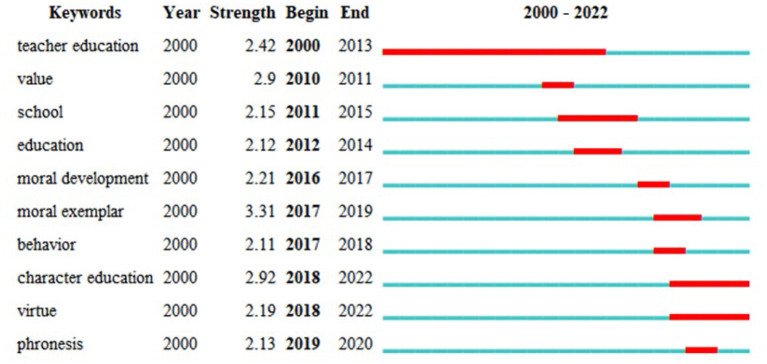
Top 10 keywords with the strongest citation bursts.

Keyword emergence and transformation can partially reflect the hot spot changes in the research field. Although the moral education development in the past decade has not produced many emerging elements, the change and transition of research hot spots still occurred in a specific period. Therefore, this paper utilizes the CiteSpace function of Burst detection to detect the top-10 keyword bursts (as shown in Figure 5) to systematically show the changes in this research topic.

Figure 5 shows that, at the beginning of the 21^3^ century, few research hot spots had high intensity, and the research focus was mainly on teacher education. This subtopic is explained in detail in the Keyword Evolutionary Analysis (see Subsection 5.2). Beginning in 2016, the field's research hot spots in the field of moral education frequently changed, and Figure 5 shows that many keyword bursts with profound connotations emerged from 2016 to 2019. For example, from 2017 to 2019, many scholars began to pay attention to the role of moral exemplars in moral education (Hamilton and LaVoi, 2017; Han et al., 2017, 2018b; Engelen et al., 2018; Nielsen, 2019; Tachibana, 2019). Character education and virtue also became the research hot spots of moral education between 2018 and 2022 (Bernal Guerrero et al., 2019; Chi-Kin Lee et al., 2021). These keywords formed a new trend that promoted the development of moral education in a deeper and more detailed direction. Research on moral education and virtue is often closely related to virtue ethics and character development, inspired by Aristotle. Therefore, the emergence of these hot spots reflects scholarly interest in the origin of morality study. Hence, Darnell et al. (2019) suggested the necessity to take seriously the increasing interest in Aristotle-inspired virtue ethics and character development within the social sciences.

In addition, phronesis is an ancient concept developed by Aristotle, and much of its discussion takes place in the sixth volume of his work, *Nicomachean Ethics*. Its intuitive meaning is practical wisdom, but understanding its meaning first requires a deep understanding of Aristotle's philosophy. Aristotle believed that human beings had both rational and irrational sides, and to have phronesis required adjusting or tailoring the irrational side of human beings to make them more rational (Darnell et al., 2019; Osman, 2019). Thus, phronesis should be distinguished from mere clever-ness. Darnell et al. (2019) noted that Aristotle's description of phronesis implied elements of the category of natural virtues such as honesty, kindness, consideration, and compassion and was similar to the neo-Kohlbergian concept of “moral judgment”, that is, the ability to weigh or adjudicate the relative priority of virtues in complex, problematic situations.

The keyword-burst analysis identified a significant revival of Aristotle's philosophy of moral education. This result confirms the present value of classical moral philosophy, in sharp contrast with the decline of Kohlberg's moral education paradigm (Kristjánsson, 2017). This contrast is a problem worth the pondering of all moral education scholars. In the past two decades of the moral education research, few research paradigms have been introduced that appear universal and in line with the needs of the times. The philosophical discussions of and theoretical research on moral education have fallen into a strange circle, as Kristjánsson wrote in a 2021 editorial. Apparently, no major, new academic trends have emerged—like Athena from the forehead of Zeus—in the past 3 years. Despite this fact, Kristjánsson (2021) remained hopeful about the future, waiting for the owl of Minerva to take her flight at dusk.

## 6. Conclusions, limitations, and future research implications

### 6.1. Conclusions

Based on a careful review of and reflection upon the research field of moral education, this paper reorganizes the theoretical connotation of moral education under the framework of virtue ethics. The reasoning follows the value judgment of Aristotle's *Nicomachean Ethics* that virtue is a kind of good quality based on acts and habits, and that it is distinguished from intellectual virtue (Ameriks and Clarke, 2000). Therefore, moral education uses various forms and systematic teaching designs (such as the establishment of moral models) to help form, cultivate, and maintain this kind of good quality through certain practices and guidance.

The evolution of any research field, including moral education, is a dynamic development process. Therefore, it is necessary to recognize, explain and analyze it from a dynamic perspective to understand the reasons for its evolution. Through a keyword performance analysis and a relevant keyword visual analysis, the following conclusions are obtained in this paper:

1. This paper systematically analyzes the scientific productivity of authors, organizations, and countries. The highly productive authors include Han and Kristjánsson, and the highly productive organizations include the University of Birmingham and Stanford University. This paper also shows the top-four countries with the largest published article volume and with close cooperation in this field (i.e., USA, United Kingdom, China, and the Netherlands), and summarizes and comments on their research focuses.
2. This paper also pays attention to the core journals (e.g., *Journal of Moral Education* etc.) and highly cited articles in the field. Additionally, it examines the scope of moral education at the academic level through the discipline categories to which the core journals belong and the key elements of the differentiation and analysis of highly cited articles. Finally, it analyzes the discipline categories related to moral education.
3. Through a cluster analysis, this paper outlines the macro-disciplinary structure of the moral education research topics, identifies the schools represented by the three clusters and their specific subject concerns, and presents different prospects for moral education as an interdisciplinary topic in various disciplines.
4. An evolutionary analysis presents the development trend of moral education over the past 20+ years. Combined with the keyword-burst analysis, this paper finds that the discipline structure has tended to be stable in the past 10 years. The classical philosophical trend represented by Aristotle has reemerged as a hot topic in the study of moral education in recent years, but the decline of some classic research paradigms has caused the discipline's development to enter a slow period.

A comprehensive review of moral education can reveal the problems existing in the current development and the direction that scholars in this field should actively explore. Firstly, the biggest gap in the current moral education research is the lack of a systematic paradigm to guide the discipline's development and to standardize its construction of a system, which is consistent with Kristjánsson (2021) viewpoints. At present, moral education is in urgent need of a disciplinary paradigm that stands on a solid theoretical basis and can keep pace with the times. A reasonable paradigm is also key to solving the problem of the slow development and evolution of moral education that was criticized by Krettenauer (2021). Secondly, more education continues to have some unsolved cross-century problems, such as the question raised by MacIntyre: whether it is possible to build a common public moral education system in the current pluralistic society (MacIntyre, 1999). Such questions have not been unanimously recognized by the academic community after more than 20 years and are not rare (Kristjánsson, 2017). Lastly, the overall review of moral education reveals that the discipline system of moral education spans positive psychology, ethics, education, and other disciplines. However, the current research all falls under a certain discourse system that analyzes moral phenomena and problems. Meanwhile, few scholars are trying to break through the disciplinary barriers of moral education or are looking for consistency among the research elements involved in the different disciplines of moral education. Future studies could try to build the multi-disciplinary thematic imagery behind moral education and construct a discourse system of universal significance for it.

### 6.2. Limitations and future research

This paper has some limitations because of some objective factors. Firstly, the bibliometric analytical software has high data standards and specifications. Therefore, to ensure the quality and integrity of the collected data, only journal articles from the SSCI of the Web of Science core collection were selected, and indexes such as the Science Citation Index Expanded (SCIE), Conference Proceedings Citation Index–Social Sciences & Humanities (CPCI-SSH) and Conference Proceedings Citation Index–Science (CPCI-S) were excluded to avoid excessive noise, which inevitably leads to the problem that the analytical data are not comprehensive. Secondly, quantitative analyses require data analyses and interpretation, which requires researchers to have a deep and comprehensive understanding of this field. Although we make efforts to overcome the adverse influence caused by personal subjective factors, some subjective color inevitably remains. To overcome these limitations, in a future study, we will expand the scope of the literature filtering, learn more widely the trends and hot topics of the moral education research, actively contact the field's scholars, and acquire objective and cutting-edge insights in the field. These efforts will greatly reduce the adverse impacts of personal subjectivity on the research and analyses.

As society dynamically evolves, technological changes will place new requirements on moral education, making it an enduring issue. This paper summarizes the main research themes of moral education research topics through systematic scientific research methods while reviewing the problems and current situation in this issue's development process. In addition, based on the research analysis, this paper puts forward some academic questions worthy of further analysis, such as why the rapid development of the moral research has failed to promote its prosperity and how to break through the strange circle of the fuzzy moral education research paradigm. Limited by its length, this paper also contains some content that has not yet been proven, including that the research methods commonly used in the field of moral education are neither classified nor quantified. Future research efforts should be made to extract quantitative information that is more comprehensive and to obtain conclusions that are more precise, which will provide interpretations that are more valuable on the development of the moral education research.

## Data availability statement

The raw data supporting the conclusions of this article will be made available by the authors, without undue reservation.

## Author contributions

Conceptualization and writing—original draft preparation: JC and YL. Methodology: YL, JD, and CW. Software: CW and JD. Writing—review and editing: JC and JD. Visualization: YL and CW. Supervision, project administration, and funding acquisition: JC. All authors read and approved the final manuscript.

## References

[B1] Althof W. Berkowitz M. W. (2006). Moral education and character education: their relationship and roles in citizenship education. J. Moral Educ. 35, 495–518. 10.1080/03057240601012204

[B2] Alvey J. E. (2001). Moral education as a means to human perfection and social order: Adam Smith's view of education in commercial society. Hist. Hum. Sci. 14, 1–18. 10.1177/09526950122120934

[B3] Ameriks K. Clarke D. M. (2000). Aristotle: Nicomachean Ethics. Cambridge: Cambridge University Press.

[B4] Aron I. E. (1977). Moral philosophy and moral education: a critique of Kohlberg's theory. School Rev. 85, 197–217. 10.1086/443328

[B5] Asif T. Guangming O. Haider M. A. Colomer J. Kayani S. Amin N. (2020). Moral education for sustainable development: comparison of university teachers' perceptions in China and Pakistan. Sustainability 12, 3014. 10.3390/su12073014

[B6] Banks J. A. Cookson P. Gay G. Hawley W. D. Irvine J. J. Nieto S.. (2001). Diversity within unity: essential principles for teaching and learning in a multicultural society. Phi Delta Kappan 83, 196–203. 10.1177/003172170108300309

[B7] Bernal Guerrero A. Gozalvez Perez V. Burguet Arfelis M. (2019). Ethical reconstruction of citizenship: a proposal between the intimate self and the public sphere. J. Moral Educ. 48, 483–498. 10.1080/03057240.2018.1563880

[B8] Berreth D. Berman S. (1997). The moral dimensions of schools. Educ. Leadership 54, 24–27.

[B9] Bielskis A. (2011). Virtue and politics: an Aristotelian reading of Niccolò Machiavelli. Problemos 80, 7–18. 10.15388/Problemos.2011.0.1311

[B10] Bleazby J. (2020). Fostering moral understanding, moral inquiry and moral habits through philosophy in schools: a Deweyian analysis of Australia's ethical understanding curriculum. J. Curric. Stud. 52, 84–100. 10.1080/00220272.2019.1650116

[B11] Bock T. Giebel H. Hazelbaker T. Tufte L. (2021). Integrating Thomistic virtue ethics with an Eriksonian identity perspective: a new moral identity assessment. J. Moral Educ. 50, 185–201. 10.1080/03057240.2019.1691511

[B12] Brabeck M. M. Rogers L. (2000). Human rights as a moral issue: lessons for moral educators from human rights work. J. Moral Educ. 29, 167–182. 10.1080/713679341

[B13] Cantero F. G. (2008). Ciudadanía y humanidad. La educación en el disenso. Teor. Educ. 20, 25–44. 10.14201/982

[B14] Carr D. (2006). The moral roots of citizenship: reconciling principle and character in citizenship education. J. Moral Educ. 35, 443–456. 10.1080/03057240601012212

[B15] Carr D. (2014). Metaphysics and methods in moral enquiry and education: some old philosophical wine for new theoretical bottles. J. Moral Educ. 43, 500–515. 10.1080/03057240.2014.943167

[B16] Chang C. M. Hung M. L. Lu J. L. Chou C. (2018). The virtues of Taiwanese internet-using adolescents: the development and validation of the Cyber Virtues Scale. J. Educ. Techno. Soc. 21, 104–111. 10.1037/t69726-000

[B17] Chen G. Xiao L. (2016). Selecting publication keywords for domain analysis in bibliometrics: a comparison of three methods. J. Informetr. 10, 212–223. 10.1016/j.joi.2016.01.006

[B18] Chen J. Wang C. Tang Y. (2022). Knowledge mapping of volunteer motivation: a bibliometric analysis and cross-cultural comparative study. Front. Psychol. 13:883150. 10.3389/fpsyg.2022.883150 35719581 PMC9204154

[B19] Cheung C. Lee T. (2010). Contributions of moral education lectures and moral discussion in Hong Kong secondary schools. Soc. Psychol. Educ. 13, 575–591. 10.1007/s11218-010-9127-x

[B20] Cheung C. K. (2007). The teaching of moral education through media education. Asia-Pac. Educ. Res. 16, 61–72. 10.3860/taper.v16i1.92

[B21] Chi-Hou C. (2004). Moral and civic education–the hidden curriculum in Macau. J. Moral Educ. 33, 553–573. 10.1080/0305724042000315707

[B22] Chi-Kin Lee J. Wong K. L. Kong R. H. M. (2021). Secondary school teachers' self-efficacy for moral and character education and its predictors: a Hong Kong perspective. Teach. Teach. 27, 32–47. 10.1080/13540602.2021.1920907

[B23] Chouliaraki L. (2008). The media as moral education: Mediation and action. Media Cult. Soc. 30, 831–852. 10.1177/016344370809609625397637

[B24] Covell K. Howe R. B. (2001). Moral education through the 3 Rs: rights, respect and responsibility. J. Moral Educ. 30, 29–41. 10.1080/03057240120033794

[B25] Dahlbeck J. (2017). A Spinozistic model of moral education. Stud. Philos. Educ. 36, 533–550. 10.1007/s11217-016-9530-7

[B26] Dahlin B. (2010). A state-independent education for citizenship? Comparing beliefs and values related to civic and moral issues among students in Swedish mainstream and Steiner Waldorf schools. J. Beliefs Values-Stud. Relig. Educ. 31, 165–180. 10.1080/13617672.2010.503629

[B27] Darnell C. Gulliford L. Kristjánsson K. Paris P. (2019). Phronesis and the knowledge-action gap in moral psychology and moral education: a new synthesis? Hum. Dev. 62, 101–129. 10.1159/000496136

[B28] Dewey J. (1959). Moral Principles in Education. New York, NY: Philosophical Library.

[B29] Ding X. Yang Z. (2022). Knowledge mapping of platform research: a visual analysis using VOSviewer and CiteSpace. Electron. Commer. Res. 22, 787–809. 10.1007/s10660-020-09410-736389800

[B30] Doyle D. P. (1997). Education and character: a conservative view. Phi Delta Kappan 78, 440–443.

[B31] Engelen B. Thomas A. Archer A. van de Ven N. (2018). Exemplars and nudges: combining two strategies for moral education. J. Moral Educ. 47, 346–365. 10.1080/03057240.2017.1396966

[B32] Fallona C. (2000). Manner in teaching: a study in observing and interpreting teachers' moral virtues. Teach. Teach. Educ. 16, 681–695. 10.1016/S0742-051X(00)00019-6

[B33] Fenstermacher G. D. (2001). On the concept of manner and its visibility in teaching practice. J. Curric. Stud. 33, 639–653. 10.1080/00220270110049886

[B34] Ferrero I. Sison A. J. G. (2014). A quantitative analysis of authors, schools and themes in virtue ethics articles in business ethics and management journals (1980-2011). Bus. Ethics 23, 375–400. 10.1111/beer.12057

[B35] Gilead T. (2011). Countering the vices: on the neglected side of character education. Stud. Philos. Educ. 30, 271–284. 10.1007/s11217-011-9223-1

[B36] Gilligan C. Attanucci J. (1988). Two moral orientations: gender differences and similarities. Merrill-Palmer Q. 34, 223–237.

[B37] Halstead M. (2004). An Islamic concept of education. Comp. Educ. 40, 517–529. 10.1080/0305006042000284510

[B38] Hamilton M. G. B. LaVoi N. M. (2017). Ethical professional identity and the development of moral exemplar collegiate coaches. J. Moral Educ. 46, 114–128. 10.1080/03057240.2017.1313724

[B39] Han H. Dawson K. J. (2022). Improved model exploration for the relationship between moral foundations and moral judgment development using Bayesian model averaging. J. Moral Educ. 51, 204–218. 10.1080/03057240.2020.1863774

[B40] Han H. Kim J. Jeong C. Cohen G. L. (2017). Attainable and relevant moral exemplars are more effective than extraordinary exemplars in promoting voluntary service engagement. Front. Psychol. 8:283. 10.3389/fpsyg.2017.00283 28326045 PMC5339280

[B41] Han H. Park J. Thoma S. J. (2018a). Why do we need to employ Bayesian statistics and how can we employ it in studies of moral education?: with practical guidelines to use JASP for educators and researchers. J. Moral Educ. 47, 519–537. 10.31234/osf.io/mruz9

[B42] Han H. Park S. C. Kim J. Jeong C. Kunii Y. Kim S. (2018b). A quantitative analysis of moral exemplars presented in moral education textbooks in Korea and Japan. Asia Pac. J. Educ. 38, 62–77. 10.1080/02188791.2018.1423950

[B43] Han H. Workman C. I. May J. Scholtens P. Dawson K. J. Glenn A. L.. (2022). Which moral exemplars inspire prosociality? Philos. Psychol. 7, 943–970. 10.1080/09515089.2022.2035343 36466108 PMC9718423

[B44] Hand M. (2019). Moral education and the justification of basic moral standards: replies to Clayton, Stevens and D'Olimpio. J. Moral Educ. 48, 529–539. 10.1080/03057240.2019.1626704

[B45] Hardy S. A. Carlo G. (2005). Identity as a source of moral motivation. Hum. Dev. 48, 232–256. 10.1159/000086859

[B46] Higgins-D'Alessandro A. (2011). Dancing up a spiral staircase: learning how best practices and policies intertwine lifelong moral development with education. J. Moral Educ. 40, 397–405. 10.1080/03057240.2011.596343

[B47] Hoekema D. A. (2010). Is there an ethicist in the house? How can we tell?, in Debating Moral Education. eds. Kiss E. Euben J. P. (Durham, NC: Duke University Press), 249–266. 10.2307/j.ctv11cw7pw.17

[B48] Hsu Y. C. Ho H. N. J. Tsai C. C. Hwang G. J. Chu H. C. Wang C. Y.. (2012). Research trends in technology-based learning from 2000 to 2009: a content analysis of publications in selected journals. J. Educ. Techno. Soc. 15, 354–370. Available online at: https://www.jstor.org/stable/jeductechsoci.15.2.354 (accessed December 14, 2022).

[B49] Husu J. Tirri K. (2003). A case study approach to study one teacher's moral reflection. Teach. Teach. Educ. 19, 345–357. 10.1016/S0742-051X(03)00019-2

[B50] Hwang G. J. Tsai C. C. (2011). Research trends in mobile and ubiquitous learning: a review of publications in selected journals from 2001 to 2010. Br. J. Educ. Technol. 42, E65–E70. 10.1111/j.1467-8535.2011.01183.x

[B51] Jie L. Desheng G. (2004). New directions in the moral education curriculum in Chinese primary schools. J. Moral Educ. 33, 495–510. 10.1080/0305724042000315617

[B52] Jordan K. Kristjánsson K. (2017). Sustainability, virtue ethics, and the virtue of harmony with nature. Environ. Educ. Res. 23, 1205–1229. 10.1080/13504622.2016.1157681

[B53] Katayama K. (2003). Is the virtue approach to moral education viable in a plural society? J. Philos. Educ. 37, 325–338. 10.1111/1467-9752.00329

[B54] Kohlberg L. (1969). Stage and sequence: the cognitive-developmental approach to socialization, in Handbook of Socialization: Theory and Research. ed. Goslin D. A. (Chicago, IL: Rand McNally), 347–480.

[B55] Kohlberg L. (1973). The claim to moral adequacy of a highest stage of moral judgment. J. Philos. 70, 630–646. 10.2307/2025030

[B56] Kohlberg L. (1981). The Philosophy of Moral Development. San Francisco, CA: Harper and Row.

[B57] Krettenauer T. (2021). Moral sciences and the role of education. J. Moral Educ. 50, 77–91. 10.1080/03057240.2020.1784713

[B58] Kristjánsson K. (2006). Emulation and the use of role models in moral education. J. Moral Educ. 35, 37–49. 10.1080/03057240500495278

[B59] Kristjánsson K. (2013). There is something about Aristotle: the pros and cons of Aristotelianism in contemporary moral education. J. Philos. Educ. 48, 48–68. 10.1111/1467-9752.12047

[B60] Kristjánsson K. (2014). Undoing bad upbringing through contemplation: an Aristotelian reconstruction. J. Moral Educ. 43, 468–483. 10.1080/03057240.2014.902809

[B61] Kristjánsson K. (2017). Moral education today: ascendancy and fragmentation. J. Moral Educ. 46, 339–346. 10.1080/03057240.2017.1370209

[B62] Kristjánsson K. (2020). Aristotelian character friendship as a “method” of moral education. Stud. Philos. Educ. 39, 349–364. 10.1007/s11217-020-09717-w

[B63] Kristjánsson K. (2021). Awaiting the owl of Minerva: some thoughts on the present and future of moral education. J. Philos. Educ. 50, 115–121. 10.1080/03057240.2021.1907074

[B64] Kuhn D. (1976). Short-term longitudinal evidence for the sequentiality of Kohlberg's early stages of moral judgment. Dev. Psychol. 12, 162–166. 10.1037/0012-1649.12.2.162

[B65] Kumashiro K. K. (2000). Toward a theory of anti-oppressive education. Rev. Educ. Res. 70, 25–53. 10.3102/00346543070001025

[B66] Kuther T. L. Higgins-D'Alessandro A. N. N. (2000). Bridging the gap between moral reasoning and adolescent engagement in risky behavior. J. Adolesc. 23, 409–422. 10.1006/jado.2000.0328 10936014

[B67] Lee A. C. M. (2022). The JME's 50-year contribution to moral education: a content analysis 1971-2021. J. Moral Educ. 51, 117–138. 10.1080/03057240.2022.2055533

[B68] Lee W. O. Ho C. H. (2005). Ideopolitical shifts and changes in moral education policy in China. J. Moral Educ. 34, 413–431. 10.1080/03057240500410160

[B69] Leihy P. Salazar J. M. (2016). The moral dimension in Chilean higher education's expansion. High. Educ. 74, 147–161. 10.1007/s10734-016-0034-8

[B70] Lewis C. J. (2018). Vygotsky and moral education: a response to and expansion of Tappan. Educ. Philos. Theory 51, 41–50. 10.1080/00131857.2018.1427576

[B71] Li X. Li H. Huang C. Yao Q. Zhao X. Chen X.. (2017). Internet news dissemination on ideological and moral cultivation in higher school. EURASIA J. Mathem. Sci. Technol. Educ. 13, 7049–7055. 10.12973/ejmste/78729

[B72] Lickona T. (1976). Critical issues in the study of moral development and behavior, in Moral Development and Behavior: Theory, Research, and Social Issues. ed. Lickona T. (New York, NY: Holt, Rinehart and Winston) 3–27.

[B73] Lickona T. (1999). Religion and character education. Phi Delta Kappan 81, 21–27.

[B74] Lin Z. Lu H. (2020). In search of a moral standard: debates over ethics education and religion in Meiji Japan. Hist. Educ. 49, 38–56. 10.1080/0046760X.2019.1651905

[B75] MacIntyre A. (1999). Social structures and their threats to moral agency. Philosophy 74, 311–329. 10.1017/S0031819199000431

[B76] MacIntyre A. Dunne J. (2002). Alasdair MacIntyre on education: in dialogue with Joseph Dunne. J. Philos. Educ. 36, 1–19. 10.1111/1467-9752.00256

[B77] Marshall J. C. Caldwell S. D. Foster J. (2011). Moral education the CHARACTER plus Way®. J. Moral Educ. 40, 51–72. 10.1080/03057240.2011.541770

[B78] McInerny R. M. (1997). Ethica Thomistica: the moral philosophy of Thomas Aquinas. Washington, DC: The Catholic University of America Press.

[B79] Merigó J. M. Gil-Lafuente A. M. Yager R. R. (2015). An overview of fuzzy research with bibliometric indicators. Appl. Soft. Comput. 27, 420–433. 10.1016/j.asoc.2014.10.035

[B80] Mischel T. (1971). Cognitive Development and Epistemology. New York, NY: Academic Press.

[B81] Motos C. R. (2010). The controversy over civic education in Spain. Eur. Polit. Sci. 9, 269–279. 10.1057/eps.2010.9

[B82] Nakazawa Y. M. (2018). Iris Murdoch's critique of three dualisms in moral education. J. Philos. Educ. 52, 397–411. 10.1111/1467-9752.12293

[B83] Nielsen C. F. (2019). Exemplarity, expressivity, education. J. Moral Educ. 48, 381–392. 10.1080/03057240.2018.1563879

[B84] Noddings N. (2010). Moral education in an age of globalization. Educ. Philos. Theory 42, 390–396. 10.1111/j.1469-5812.2008.00487.x

[B85] Nucci L. Turiel E. (2009). Capturing the complexity of moral development and education. Mind Brain Educ. 3, 151–159. 10.1111/j.1751-228X.2009.01065.x

[B86] Osman Y. (2019). The significance in using role models to influence primary school children's moral development: pilot study. J. Moral Educ. 48, 316–331. 10.1080/03057240.2018.1556154

[B87] Pan X. Yan E. Cui M. Hua W. (2018). Examining the usage, citation, and diffusion patterns of bibliometric mapping software: a comparative study of three tools. J. Informetr. 12, 481–493. 10.1016/j.joi.2018.03.005

[B88] Pantić N. Wubbels T. (2012). The role of teachers in inculcating moral values: operationalisation of concepts. J. Beliefs Values-Stud. Relig. Educ. 33, 55–69. 10.1080/13617672.2012.650030

[B89] Persson I. Savulescu J. (2013). Getting moral enhancement right: the desirability of moral bioenhancement. Bioethics 27, 124–131. 10.1111/j.1467-8519.2011.01907.x 21797913 PMC3378470

[B90] Peters R. S. (2015). Moral Development and Moral Education. London: Routledge.

[B91] Piaget J. (1932). The Moral Judgment of the Child. New York, NY: Free Press.

[B92] Price D. J. D. S. (1963). Little Science, Big Science. Warrenton, OSU: Columbia University Press. 10.7312/pric91844

[B93] Ranson S. (2000). Recognizing the pedagogy of voice in a learning community. Educ. Manag. Adm. 28, 263–279. 10.1177/0263211X000283003

[B94] Reiman A. J. Dotger B. H. (2008). What does innovation mean for moral educators? J. Moral Educ. 37, 151–164. 10.1080/03057240802009124

[B95] Rest J. Thoma S. Edwards L. (1997). Designing and validating a measure of moral judgment: stage preference and stage consistency approaches. J. Educ. Psychol. 89, 5–28. 10.1037/0022-0663.89.1.5

[B96] Richmond A. S. Cummings R. (2004). In support of the cognitive-developmental approach to moral education: a response to David Carr. J. Moral Educ. 33, 197–205. 10.1080/0305724042000215230

[B97] Ronkainen R. Kuusisto E. Eisenschmidt E. Tirri K. (2021). Ethical sensitivity of Finnish and Estonian teachers. J. Moral Educ. 50, 1–17. 10.1080/03057240.2021.1960491

[B98] Sanger M. N. Osguthorpe R. D. (2011). Teacher education, preservice teacher beliefs, and the moral work of teaching. Teach. Teach. Educ. 27, 569–578. 10.1016/j.tate.2010.10.011

[B99] Santas A. (2000). Teaching anti-racism. Stud. Philos. Educ. 19, 349–361. 10.1023/A:1005298916161

[B100] Sarid A. (2012). Between thick and thin: Responding to the crisis of moral education. J. Moral Educ. 41, 245–260. 10.1080/03057240.2012.678054

[B101] Schuitema J. Dam G. T. Veugelers W. (2008). Teaching strategies for moral education: a review. J. Curric. Stud. 40, 69–89. 10.1080/00220270701294210

[B102] Seroczynski A. D. (2015). Virtues and vices in positive psychology: a philosophical critique. J. Moral Educ. 44, 112–114. 10.1080/03057240.2014.969886

[B103] Shao X. Wang H. (2017). Impact of we media on deconstruction and reconstruction of college students' ideological and moral outlook. EURASIA J. Mathem. Sci. Technol. Educ. 13, 8113–8119. 10.12973/ejmste/80772

[B104] Sin W. (2022). Modesty, Confucianism, and active indifference. Educ. Philos. Theory 54, 1–11. 10.1080/00131857.2022.2082939

[B105] Sison A. J. G. Redín D. M. (2022). If MacIntyre ran a business school… how practical wisdom can be developed in management education. Bus. Ethics Environ. Resp. 32, 274–291. 10.1111/beer.12471

[B106] Slote M. (2016). Moral self-cultivation east and west: a critique. J. Moral Educ. 45, 192–206. 10.1080/03057240.2016.1174674

[B107] Solomon D. Watson M. S. Battistich V. A. (2001). Teaching and schooling effects on moral/prosocial development, in Handbook of Research on Teaching. ed. Richardson V. (Washington, DC: American Educational Research Association), 566–603.

[B108] Stanley F. (2003). Save the world on your own time. Available online at: https://www.chronicle.com/article/save-the-world-on-your-own-time/ (accessed October 2, 2022).

[B109] Stanley F. (2004). The case for academic autonomy. Available online at: https://www.chronicle.com/article/the-case-for-academic-autonomy/ (accessed October 2, 2022).

[B110] Su Y. S. Lin C. L. Chen S. Y. Lai C. F. (2019). Bibliometric study of social network analysis literature. Libr. Hi Tech 38, 420–433. 10.1108/LHT-01-2019-0028

[B111] Surprenant C. W. (2010). Kant's contribution to moral education: the relevance of catechistics. J. Moral Educ. 39, 165–174. 10.1080/03057241003754898

[B112] Tachibana K. (2019). Nonadmirable moral exemplars and virtue development. J. Moral Educ. 48, 346–357. 10.1080/03057240.2019.1577723

[B113] Tai Wei T. Lee Chin C. (2004). Moral and citizenship education as statecraft in Singapore: a curriculum critique. J. Moral Educ. 33, 597–606. 10.1080/0305724042000315644

[B114] Tan C. (2020). Digital confucius? Exploring the implications of artificial intelligence in spiritual education. Connect. Sci. 32, 280–291. 10.1080/09540091.2019.1709045

[B115] Tappan M. B. (1997). Language, culture, and moral development: a Vygotskian perspective. Dev. Rev. 17, 78–100. 10.1006/drev.1996.0422

[B116] Thornberg R. (2008). The lack of professional knowledge in values education. Teach. Teach. Educ. 24, 1791–1798. 10.1016/j.tate.2008.04.004

[B117] Verducci S. (2014). Self-doubt: one moral of the story. Stud. Philos. Educ. 33, 609–620. 10.1007/s11217-014-9407-6

[B118] Villenas S. (2001). Latina mothers and small-town racisms: creating narratives of dignity and moral education in North Carolina. Anthropol. Educ. Q. 32, 3–28. 10.1525/aeq.2001.32.1.3

[B119] Wang C. L. Dai J. Xu L. J. (2022). Big data and data mining in education: a bibliometrics study from 2010 to 2022, in Proceedings of 2022 7th International Conference on Cloud Computing and Big Data Analytics (Chengdu: IEEE), 507–512. 10.1109/ICCCBDA55098.2022.9778874

[B120] Wansheng Z. Wujie N. (2004). The moral education curriculum for junior high schools in 21st century China. J. Moral Educ. 33, 511–532. 10.1080/0305724042000327993

[B121] Wanxue Q. Hanwei T. (2004). The social and cultural background of contemporary moral education in China. J. Moral Educ. 33, 465–480. 10.1080/0305724042000315590

[B122] Wardekker W. (2004). Moral education and the construction of meaning. Educ. Rev. 56, 183–192. 10.1080/0031910410001693263

[B123] Wardekker W. L. (2001). Schools and moral education: conformism or autonomy? J. Philos. Educ. 35, 101–114. 10.1111/1467-9752.00212

[B124] Wong M. Y. (2020). University students' perceptions of learning of moral education: a response to lifelong moral education in higher education. Teach. High Educ. 25, 1–18. 10.1080/13562517.2020.1852201

[B125] Woods M. (2005). Nursing ethics education: are we really delivering the good (s)? Nurs. Ethics 12, 5–18. 10.1191/0969733005ne754oa 15685964

[B126] Xiaoman Z. Cilin L. (2004). Teacher training for moral education in China. J. Moral Educ. 33, 481–494. 10.1080/0305724042000315608

[B127] Yadegaridehkordi E. Noor N. F. B. M. Ayub M. N. B. Affal H. B. Hussin N. B. (2019). Affective computing in education: a systematic review and future research. Comput. Educ. 142, 103649. 10.1016/j.compedu.2019.103649

[B128] Zhang Q. Saharuddin N. B. Aziz N. A. B. A. (2022). The analysis of teachers' perceptions of moral education curriculum. Front. Psychol. 13, 967927. 10.3389/fpsyg.2022.967927 35959034 PMC9362598

