# Supplementary material for: Development and status of moral education research: Visual analysis based on knowledge graph
Source: Front Psychol. 2023 Jan 4;13:1079955. doi: 10.3389/fpsyg.2022.1079955 (PMC9846641; doi:10.3389/fpsyg.2022.1079955)
Supplement: Supplementary file 1 [file Table_1.docx]

Supplementary Material

# Supplementary Figures and Tables

**Appendix 1** Top 100 highly-cited articles.

| **Rank** | **First Author** | **Title** | **Year** | **Citations** |
| --- | --- | --- | --- | --- |
| 1 | Villenas | Latina mothers and small-town racisms: creating narratives of dignity and moral education in North Carolina | 2001 | 124 |
| 2 | Halstead | An Islamic concept of education | 2004 | 110 |
| 3 | Hardy | Identity as a source of moral motivation | 2005 | 94 |
| 4 | Thornberg | The lack of professional knowledge in values education | 2008 | 84 |
| 5 | Persson | Getting moral enhancement right: the desirability of moral bioenhancement | 2013 | 84 |
| 6 | Nucci | Capturing the complexity of moral development and education | 2009 | 83 |
| 7 | Sanger | Teacher education, preservice teacher beliefs, and the moral work of teaching | 2011 | 78 |
| 8 | Chouliaraki | The media as moral education: mediation and action | 2008 | 76 |
| 9 | Noddings | Moral education in an age of globalization | 2010 | 70 |
| 10 | Kristjansson | Emulation and the use of role models in moral education | 2006 | 64 |
| 11 | Sanderse | The meaning of role modelling in moral and character education | 2013 | 64 |
| 12 | Diessner | Who engages with moral beauty? | 2013 | 61 |
| 13 | Hardy | Parenting dimensions and adolescents’ internalisation of moral values | 2008 | 56 |
| 14 | Han | Attainable and relevant moral exemplars are more effective than extraordinary exemplars in promoting voluntary service engagement | 2017 | 55 |
| 15 | Johnson | “every experience is a moving force”: identity and growth through mentoring | 2003 | 55 |
| 16 | Lee | Ideopolitical shifts and changes in moral education policy in China | 2005 | 54 |
| 17 | Warnick | A framework for professional ethics courses in teacher education | 2011 | 53 |
| 18 | Pring | Education as a moral practice | 2001 | 52 |
| 19 | Kristjansson | Ten myths about character, virtue and virtue education - plus three well-founded misgivings | 2013 | 51 |
| 20 | Willemse | Values in education: a challenge for teacher educators | 2005 | 48 |
| 21 | Verducci | A moral method? thoughts on cultivating empathy through method acting | 2000 | 48 |
| 22 | Covell | Moral education through the 3 rs: rights, respect and responsibility | 2001 | 44 |
| 23 | Carr | On the contribution of literature and the arts to the educational cultivation of moral virtue, feeling and emotion | 2005 | 41 |
| 24 | Wang | Confucian thinking in traditional moral education: key ideas and fundamental features | 2004 | 40 |
| 25 | Walker | The model and the measure: an appraisal of the Minnesota approach to moral development | 2002 | 40 |
| 26 | Maria Aviles | The abuse between equals: “bullying” | 2011 | 39 |
| 27 | Qi | The social and cultural background of contemporary moral education in China | 2004 | 38 |
| 28 | Osguthorpe | On the reasons we want teachers of good disposition and moral character | 2008 | 38 |
| 29 | Li | Deyu as moral education in modern China: ideological functions and transformations | 2004 | 37 |
| 30 | Diessner | Beauty and hope: a moral beauty intervention | 2006 | 37 |
| 31 | Moran | Can Kant have an account of moral education? | 2009 | 36 |
| 32 | Nicholson | Relational leadership for sustainability: building an ethical framework from the moral theory of ‘ethics of care’ | 2019 | 35 |
| 33 | Willemse | The moral aspects of teacher educators’ practices | 2008 | 34 |
| 34 | Le Grange | Ubuntu, ukama, environment and moral education | 2012 | 33 |
| 35 | Yu | The revival of Confucianism in Chinese schools: a historical-political review | 2008 | 33 |
| 36 | Thornberg | Teachers’ views on values education: a qualitative study in Sweden and turkey | 2013 | 33 |
| 37 | Han | Virtue ethics, positive psychology, and a new model of science and engineering ethics education | 2015 | 33 |
| 38 | Cheng | Moral education in Hong Kong: Confucian-parental, Christian-religious and liberal-civic influences | 2004 | 32 |
| 39 | Han | Purpose as a moral virtue for flourishing | 2015 | 32 |
| 40 | Walker | Towards a new era of character education in theory and in practice | 2015 | 31 |
| 41 | Willemse | Fostering teachers’ professional development for citizenship education | 2015 | 31 |
| 42 | Sanger | What we need to prepare teachers for the moral nature of their work | 2008 | 30 |
| 43 | Husu | A case study approach to study one teacher’s moral reflection | 2003 | 30 |
| 44 | Doan | Moral education or political education in the Vietnamese educational system? | 2005 | 29 |
| 45 | Bonnett | Environmental concern, moral education and our place in nature | 2012 | 29 |
| 46 | Carr | Moral values and the arts in environmental education: towards an ethics of aesthetic appreciation | 2004 | 28 |
| 47 | Resnik | Ethical virtues in scientific research | 2012 | 28 |
| 48 | Fallona | Manner in teaching: a study in observing and interpreting teachers’ moral virtues | 2000 | 28 |
| 49 | Runco | Creativity in the moral domain: integration and implications | 2003 | 28 |
| 50 | Sanger | Modeling as moral education: documenting, analyzing, and addressing a central belief of preservice teachers | 2013 | 27 |
| 51 | Englund | On moral education through deliberative communication | 2016 | 27 |
| 52 | Lee | Changes and challenges for moral education in Taiwan | 2004 | 26 |
| 53 | Reid | Sport and moral education in Plato’s republic | 2007 | 26 |
| 54 | Graham | Centripetal and centrifugal forces in the moral circle: competing constraints on moral learning | 2017 | 26 |
| 55 | Thornberg | Moral and citizenship educational goals in values education: a cross-cultural study of Swedish and Turkish student teachers’ preferences | 2016 | 26 |
| 56 | Kuther | Bridging the gap between moral reasoning and adolescent engagement in risky behavior | 2000 | 26 |
| 57 | Skoe | The relationship between empathy-related constructs and care-based moral development in young adulthood | 2010 | 26 |
| 58 | Bergman | Caring for the ethical ideal: Nel Noddings on moral education | 2004 | 25 |
| 59 | Porter | Moral and political identity and civic involvement in adolescents | 2013 | 25 |
| 60 | Sanger | Making sense of approaches to moral education | 2005 | 24 |
| 61 | Engelen | Exemplars and nudges: combining two strategies for moral education | 2018 | 24 |
| 62 | Greene | Lost convictions - debating both sides and the ethical self-fashioning of liberal citizens | 2005 | 24 |
| 63 | Davis | What’s wrong with character education? | 2003 | 23 |
| 64 | Semetsky | The folds of experience, or: constructing the pedagogy of values | 2010 | 23 |
| 65 | Payne | Challenges for research ethics and moral knowledge construction in the applied social sciences | 2000 | 23 |
| 66 | Lu | New directions in the moral education curriculum in Chinese primary schools | 2004 | 22 |
| 67 | Nucci | Integrating moral and social development within middle school social studies: a social cognitive domain approach | 2015 | 22 |
| 68 | Han | Why do we need to employ Bayesian statistics and how can we employ it in studies of moral education?: with practical guidelines to use JASP for educators and researchers | 2018 | 21 |
| 69 | Standish | Ethics before equality: moral education after Levinas | 2001 | 21 |
| 70 | Boyd | The legacies of liberalism and oppressive relations: facing a dilemma for the subject of moral education | 2004 | 21 |
| 71 | Rabin | Teaching care ethics: conceptual understandings and stories for learning | 2013 | 21 |
| 72 | Froerer | Disciplining the saffron way: moral education and the Hindu Rashtra | 2007 | 21 |
| 73 | Williams | Character education in a public high school: a multi-year inquiry into unified studies | 2003 | 21 |
| 74 | Hand | Towards a theory of moral education | 2014 | 20 |
| 75 | Brownlee | Teachers’ and children’s personal epistemologies for moral education: case studies in early years elementary education | 2012 | 20 |
| 76 | Lovett | Levels of moralisation: a new conception of moral sensitivity | 2010 | 20 |
| 77 | Merry | The relevance of cosmopolitanism for moral education | 2011 | 19 |
| 78 | Zhu | Teacher training for moral education in China | 2004 | 18 |
| 79 | Lepage | Comparing teachers’ views on morality and moral education, a comparative study in turkey and the United States | 2011 | 18 |
| 80 | Nucci | Recovering the role of reasoning in moral education to address inequity and social justice | 2016 | 18 |
| 81 | Arnold | Sport, moral development, and the role of the teacher: implications for research and moral education | 2001 | 18 |
| 82 | Drewe | The logical connection between moral education and physical education | 2000 | 18 |
| 83 | Sherman | Of manners and morals | 2005 | 18 |
| 84 | Han | Analysing theoretical frameworks of moral education through Lakatos’s philosophy of science | 2014 | 17 |
| 85 | Curren | Motivational aspects of moral learning and progress | 2014 | 17 |
| 86 | Gleeson | The teacher as moral educator: comparative study of secondary teachers in catholic schools in Australia and Ireland | 2016 | 16 |
| 87 | Maxwell | Imitation, imagination and re-appraisal: educating the moral emotions | 2005 | 16 |
| 88 | Musschenga | Moral intuitions, moral expertise and moral reasoning1 | 2009 | 16 |
| 89 | Carr | Moral education and the perils of developmentalism | 2002 | 16 |
| 90 | Ruiz | Moral education as pedagogy of alterity | 2004 | 16 |
| 91 | Carr | Moral education at the movies: on the cinematic treatment of morally significant story and narrative | 2006 | 16 |
| 92 | Williams | Felt moral obligation and the moral judgement-moral action gap: toward a phenomenology of moral life | 2012 | 16 |
| 93 | Lee | Moral education trends over 40 years: a content analysis of the journal of moral education (1971-2011) | 2013 | 15 |
| 94 | Zhan | The moral education curriculum for junior high schools in 21st century China | 2004 | 15 |
| 95 | Asif | Moral education for sustainable development: comparison of university teachers’ perceptions in China and Pakistan | 2020 | 15 |
| 96 | Splitter | Identity, citizenship and moral education | 2011 | 15 |
| 97 | Wardekker | Schools and moral education: conformism. or autonomy? | 2001 | 14 |
| 98 | Dill | Durkheim and Dewey and the challenge of contemporary moral education | 2007 | 14 |
| 99 | Gozalvez | The articulation of justice and care in moral education: from substitutive universalism to situated human rights ethics | 2016 | 14 |
| 100 | Chia | The elusive goal of nation building: Asian/Confucian values and citizenship education in Singapore during the 1980s | 2011 | 14 |
